# Supplementary material for: Effects of different roughness on Brazilian splitting characteristics of rock-concrete interface
Source: PLoS One. 2024 Aug 23;19(8):e0307190. doi: 10.1371/journal.pone.0307190 (PMC11343416; doi:10.1371/journal.pone.0307190)
Supplement: Attachment — Submitted filename: Review comments-PONE-D-23-40490.pdf [file pone.0307190.s002.pdf]

This paper (PONE-D-23-40490) explores the influence of roughness on the tensile properties of rock-concrete composite disc specimens. The sinusoidal periodic variation of crack-related parameters with roughness is an interesting observation. This paper offers valuable insights for practical applications, especially in construction projects involving rock-concrete interfaces. However, the experimental methods used in this study were simplistic and lacked depth of research. I suggest to the Editor that the manuscript requests a “major revision.” Some specific comments for improving the manuscript are given as follows.

1. Introduction: The origin and significance of the Brazilian Splitting Test in the field of rock mechanics are considered common knowledge, requiring minimal elaboration. Considering this, the author is encouraged to supplement the introduction with content directly pertinent to the research focus of this manuscript. By providing additional context on the specific relevance of the research content, such as its implications in practical engineering applications, the introduction can be enriched to better engage the reader and underscore the importance of the investigation.
2. As the author acknowledges in the Introduction, numerous scholars have employed advanced testing techniques, such as AE, DIC, and transparent materials, to observe the initiation and propagation of cracks. In contrast, this article does not leverage these sophisticated testing methods for supplementary investigation. Therefore, in comparison to existing research, what constitutes the innovative aspects of this article?
3. Sample making: Provide additional details on the specific methods employed for artificially grooving the specimens. This could include information on the tools, techniques, and parameters used in the grooving process. Suggest adding images or schematics that can illustrate the groove treatment process on the rock-concrete specimens. This can provide readers with a clearer understanding of the experimental setup.
4. Splitting energy rate: The term "energy" mentioned by the author refers to what exactly? If it pertains to elastic strain energy, is the strain limited to axial strain $\varepsilon_y$

alone, or does it also encompass horizontal strain $\varepsilon_x$? Kindly provide a more elaborate explanation of the concept of energy and the specific calculation methods.

5. Crack evolution characteristics: The author's classification of the crack propagation process solely based on the stress-strain curve appears overly simplistic. If experimental conditions permit, it is recommended to consider employing techniques such as AE or DIC for supplementary investigation. This would add depth to the research results, enhancing both the reliability and comprehensiveness of the study.
6. Discussion: What criteria does the author use to distinguish between shear failure and tensile failure? Additionally, by what means were the observations made regarding the phenomenon of stress concentration?
7. What is the purpose of Figure 7? If it aims to illustrate the influence of different roughness levels on the Brazilian Splitting fracture surface, it is recommended to supplement the analysis with a 3D Laser Scanner.
