# Supplementary material for: Effects of different roughness on Brazilian splitting characteristics of rock-concrete interface
Source: PLoS One. 2024 Aug 23;19(8):e0307190. doi: 10.1371/journal.pone.0307190 (PMC11343416; doi:10.1371/journal.pone.0307190)
Supplement: Attachment — Submitted filename: Detailed Response to Reviewers202405.docx [file pone.0307190.s003.docx]

Response to Editor and Reviewers

Dear Editor and Reviewers,

First of all, all authors would like to take this opportunity to thank you and the anonymous reviewers for the valuable comments and suggestions on our paper (Paper no: PONE-D-23-40490), Paper title: **Effects of different roughness on brazilian splitting characteristics of rock-concrete interface**). We have studied all the comments carefully and have made corrections accordingly.

The main corrections in the paper and the responses to the reviewers’ comments are given as follows.

Revisions in the text are shown using red highlight for additions, and blue highlight for grammatical checks. We have addressed all the comments one by one made by the reviewers in the enclosed files.

The manuscript has been resubmitted to the journal and we are looking forward to your decision as soon as possible

With my best regards.

Sincerely yours,

Yan Chen, Gaofei Wang, Lei Zhou, Liangtao Deng, Jiahao Wang

2024-05-20

Reviewer 1

Based on the Brazilian splitting experiment, this paper studies the effects of six different roughness on rock-concrete properties, analyzes the influence of interface roughness on the tensile strength, deformation, splitting energy rate and failure mode of rock-concrete composites. Although the test is simple, the analysis is acceptable. However, the manuscript has some problems and it would require some major revisions.

**Answer:**

Thanks very much for your kind suggestion. We deeply appreciate the comments concerning the manuscript. Revisions in the text are shown using red highlight for additions, and blue highlight for grammatical checks.

***Comment 1:*** The writing of the “1 Introduction” lacks the reference of roughness to the characteristics of coal-rock combination and suggests the author consult relevant literature and supplement them in time.

**Answer:** Revision at Page 2 Line 43~45.

Thanks very much for your kind suggestion. We have rewritten the abstract in the revised manuscript, giving a concise description of the paper and listing some new findings.

***Comment 2:*** Please introduction the significance of the effect of rock-concrete failure to the tunnel engineering.

**Answer:** Revision at Page 2 Line 33~46; Page 3 Line 47~48.

Thank you for the reviewer’s comment. We have introduced the influence of rock-concrete failure to tunnel engineering in the introduction of the revised manuscript.

***Comment 3:*** “The peak tensile strain is often smaller than that without serrations, indicating that the center has serrations” the relationship between “peek tensile strain” and “serrations” need to be further explanation.

**Answer:** Revision at Page 11 Line 207 ~ 210; Page 12 Line 211 ~ 219.

Thank you very much for your friendly advice. We have supplemented the relationship between “peek tensile strain” and “serrations” in the revised draft, and explained the reasons for this phenomenon.

***Comment 4:*** I suggest the author should increase the basic mechanical parameters of rock and concrete and increase the integrity of the paper.

**Answer:** Revision at Page 10 Line 185~192; Page 11 Line 193~196.

We really appreciate the reviewer’s suggestion. We supplemented the Brazilian splitting data of sandstone and concrete in the revised manuscript.

***Comment 5:*** The author should be given the calculation formula of crack evolution parameters.

**Answer:** Revision at Page 16 Line 303~305; Page 17 Line 306~327; Page 18 Line 328.

Thanks to the reviewer’s suggestion. We supplemented the calculation formula of crack propagation parameters in the revised manuscript.

***Comment 6:*** I suggest the author give the fitting formula of Fig.6.

**Answer:** Revision at Page 19 Line 346~347.

Thank you for the reviewer’s advice. We supplemented the fitting formula of Fig.6 in the revised manuscript.

Reviewer 2

This paper (PONE-D-23-40490) explores the influence of roughness on the tensile

properties of rock-concrete composite disc specimens. The sinusoidal periodic variation

of crack-related parameters with roughness is an interesting observation. This paper

offers valuable insights for practical applications, especially in construction projects

involving rock-concrete interfaces. However, the experimental methods used in this

study were simplistic and lacked depth of research. I suggest to the Editor that the

manuscript requests a “major revision.” Some specific comments for improving the

manuscript are given as follows.

**Answer:**

Thanks very much for your kind suggestion. We have revised the manuscript according to each review comment. Revisions in the text are shown using red highlight for additions, and blue highlight for grammatical checks.

***Comment 1:*** Introduction: The origin and significance of the Brazilian Splitting Test in the field of rock mechanics are considered common knowledge, requiring minimal elaboration. Considering this, the author is encouraged to supplement the introduction with content directly pertinent to the research focus of this manuscript. By providing additional context on the specific relevance of the research content, such as its implications in practical engineering applications, the introduction can be enriched to better engage the reader and underscore the importance of the investigation.

**Answer:** Revision at Page 2 Line 33~46; Page 3 Line 47~68; Page 4 Line 69~77.

Thanks very much for your kind suggestion. We have rewritten a new introduction, deleted the origin and significance of the Brazilian splitting test in the field of rock mechanics, and supplemented the relevant content related to the focus of this study.

***Comment 2:*** As the author acknowledges in the Introduction, numerous scholars have employed advanced testing techniques, such as AE, DIC, and transparent materials, to observe the initiation and propagation of cracks. In contrast, this article does not leverage these sophisticated testing methods for supplementary investigation. Therefore, in comparison to existing research, what constitutes the innovative aspects of this article?

**Answer:**

Thanks to the reviewer’s suggestion. Unfortunately, our laboratory lacks precision instruments such as AE, DIC, and transparent materials. Therefore, in our tests, we cannot monitor the evolution of failure. In future research, we will seek the help of others to obtain more advanced equipment to monitor the failure process of the specimen and to analyze the failure characteristics of the specimen more accurately. The innovation of this paper is to use the method of artificial groove to make rock-concrete composite disc samples with different roughness, and to quantify the roughness value of the samples under different working conditions by sand filling method, so as to study the influence of roughness on the tensile properties of rock-concrete composite disc samples.

***Comment 3:*** Sample making: Provide additional details on the specific methods employed for artificially grooving the specimens. This could include information on the tools, techniques, and parameters used in the grooving process. Suggest adding images or schematics that can illustrate the groove treatment process on the rock-concrete specimens. This can provide readers with a clearer understanding of the

experimental setup.

**Answer:** Revision at Page 5 Line 110~111; Page 6 Line 112~122; Page 8 Line 157~159; Page 9 Line 160; Page 9 Line 171~179.

Thank you for the reviewer’s advice. We do not have a detailed description of the specific methods of manual grooving samples. After verification with the sample processing party, we give a more detailed description of the tools, parameters and related technologies used in the grooving process. In the revised manuscript, we supplemented the process flow diagram of the grooving process and the related schematic diagrams of the grooving samples with different roughness, hoping to provide readers with a more concise test introduction and facilitate readers to quickly and clearly understand the experimental setup.

***Comment 4:*** Splitting energy rate: The term "energy" mentioned by the author refers to what exactly? If it pertains to elastic strain energy, is the strain limited to axial strain ε_y_ alone, or does it also encompass horizontal strain ε_x_? Kindly provide a more elaborate explanation of the concept of energy and the specific calculation methods.

**Answer:** Revision at Page 16 Line 303~305; Page 17 Line 306~327; Page 18 Line 328.

Thanks very much for your kind suggestion. We explain the energy in the newly revised manuscript, and explain the related concepts and specific calculation methods of energy in detail. The elastic strain energy designed and calculated in this paper is only related to the axial strain ε_y_.

***Comment 5:*** Crack evolution characteristics: The author's classification of the crack propagation process solely based on the stress-strain curve appears overly simplistic. If experimental conditions permit, it is recommended to consider employing techniques such as AE or DIC for supplementary investigation. This would add depth to the research results, enhancing both the reliability and comprehensiveness of the study.

**Answer:** Thanks to the reviewer’s suggestion. As mentioned in Answer 2, our laboratory lacks precision instruments such as AE, DIC, and transparent materials, so the content we discuss in this paper mainly focuses on the stress-strain curve of the sample. In future experiments, our research group will consider using more test instruments and better test conditions to do deeper research to improve the reliability and comprehensiveness of the research.

***Comment 6:*** Discussion: What criteria does the author use to distinguish between shear failure and tensile failure? Additionally, by what means were the observations made regarding the phenomenon of stress concentration?

**Answer:** Revision at Page 21 Line 366 ~ Line 370.

Thanks very much for your kind suggestion. In section 3.1.1, the calculation method of tensile strength is described. If deleted, it may affect the integrity of the paper and make readers question the calculation method of tensile strength. To that end, the narrative of the paragraph was retained in the revised manuscript. In the revised manuscript, we simplified the first paragraph of Section 3.7.

***Comment 7:*** What is the purpose of Figure 7? If it aims to illustrate the influence of different roughness levels on the Brazilian Splitting fracture surface, it is recommended to supplement the analysis with a 3D Laser Scanner.

**Answer:**

Thanks very much for your kind suggestion. Fig.10 (original Fig.7) shows the failure modes of Brazilian splitting discs with different roughness levels. This paper briefly explains the influence of roughness on the surface of Brazilian splitting fracture. Due to the limited laboratory conditions, it is temporarily impossible to carry out 3D Laser Scanner of the sample port for more profound analysis. In the future test, we will consider using a 3D Laser Scanner to scan the failure section of the sample and conduct in-depth analysis.
